# Beyond the Lungs: Atypical Systemic Presentation of Pulmonary Tuberculosis in a Young Woman

**DOI:** 10.51894/001c.165595

**Published:** 2026-07-31

**Authors:** Misbah Fatima, Mikhail Salnikov, Zohaib Khan, Khabbab Alnimer, Wasif Hafeez, Kamal Khalil

**Affiliations:** 1 DMC - Sinai Grace

## 80

### Background

Tuberculosis, caused by Mycobacterium tuberculosis, remains a major cause of infectious morbidity worldwide. Although pulmonary disease is the most common manifestation, TB may present with diverse systemic and extrapulmonary symptoms. In low-prevalence settings, atypical presentations in immunocompetent patients without traditional epidemiologic risk factors may delay diagnosis. Constitutional symptoms accompanied by gastrointestinal complaints often prompt evaluation for inflammatory, malignant, or alternative infectious conditions, obscuring the underlying diagnosis and increasing the risk of disease progression and transmission.

### Case Presentation

A 24-year-old female with a history of endometriosis and uterine fibroids presented with two days of nausea, headache, dizziness, near-syncope, and multiple episodes of non-bilious, non-bloody emesis. She reported five months of progressive unintentional weight loss, anorexia, intermittent postprandial lower abdominal pain, daily watery diarrhea, and two episodes of dark red stools six days prior to presentation. She also endorsed night fevers, night sweats, and a one-month history of productive cough with yellow sputum and dyspnea at rest. She denied recent travel, tuberculosis exposure, or sick contacts. On presentation, she was tachycardic but normotensive, afebrile, and saturating well on room air. Laboratory studies showed mild hyponatremia, thrombocytosis, hypoalbuminemia, elevated inflammatory markers, and microcytic anemia consistent with iron deficiency. Chest radiography revealed bilateral airspace disease with a cavitary lesion in the right upper lobe. CT pulmonary angiography excluded pulmonary embolism but demonstrated right upper lobe consolidation, bronchiectasis, multiple cavitary lesions (largest 3.9 × 5.1 cm), and diffuse bilateral tree-in-bud nodularity suggestive of endobronchial spread. Abdominal CT showed mild mesenteric lymphadenopathy and duodenal dilation concerning for superior mesenteric artery syndrome. An extensive infectious, autoimmune, and malignancy workup was initiated. Sputum acid-fast bacilli smear returned positive, confirming pulmonary tuberculosis. The patient was placed in airborne isolation, and public health authorities were notified. Although she initially left the hospital against medical advice, she was later located by public health services and started on directly observed therapy.

### Conclusion

This case highlights how systemic and gastrointestinal symptoms can obscure the diagnosis of tuberculosis, even in young immunocompetent patients without traditional risk factors. Early recognition and prompt isolation remain essential to prevent disease progression and limit community transmission.

